# Knowledge of chronic complications of diabetes among persons living with type 2 diabetes mellitus in northern Ghana

**DOI:** 10.1371/journal.pone.0241424

**Published:** 2020-10-28

**Authors:** Richard Adongo Afaya, Victoria Bam, Thomas Bavo Azongo, Agani Afaya

**Affiliations:** 1 Department of Surgery, Tamale West Hospital, Tamale, Ghana; 2 Department of Nursing, Faculty of Allied Health Sciences, Kwame Nkrumah University of Science and Technology, Kumasi, Ghana; 3 Department of Public Health, School of Allied Health Sciences, University for Development Studies, Tamale, Ghana; 4 Department of Nursing, School of Nursing and Midwifery, University of Health and Allied Sciences, Ho, Ghana; 5 College of Nursing, Yonsei University, Seoul, Republic of South Korea; Ministry of Health and Sports, MYANMAR

## Abstract

**Introduction:**

Diabetes mellitus is a complex disease that affects many organ systems, leading to concerns about deteriorating population health status and ever-increasing healthcare expenditure. Many people with diabetes do not achieve optimal glycaemic control and other metabolic indices, leading to a heightened risk of developing complications. Adequate knowledge of diabetes complications is a prerequisite for risk-factor reduction and prevention of the consequences of the disease. Therefore, this study aimed to evaluate the knowledge of chronic complications of diabetes among persons living with type 2 diabetes mellitus in northern Ghana.

**Method:**

A descriptive cross-sectional study was conducted among 320 patients with type 2 diabetes mellitus in northern Ghana. The consecutive sampling technique was employed to recruit participants from September to November 2018. Data analysis was performed using IBM statistical package for social science version 23. Descriptive statistics such as frequencies and percentages were used. Both bivariate and multivariate logistic regression analysis were employed to determine associations between knowledge of diabetes complications and demographic/clinical characteristics of participants, at 95% confidence interval with statistical significance at *P*<0.05.

**Results:**

The majority of participants (54.1%) had inadequate knowledge and 45.9% had adequate knowledge of diabetes complications. The factors associated with inadequate level of knowledge were female gender [AOR = 0.29 (95%CI: 0.14–0.56), p<0.001], older age [AOR = 0.45 (95%CI:0.20–0.99), p = 0.049], primary education [AOR = 0.13 (95%CI: 0.03–0.51), p = 0.004], no formal education [AOR = 0.16 (95%CI: 0.05–0.50), p = 0.002], rural dwellers [AOR = 0.50 (95%CI: 0.27–0.95), p = 0.033] and unknown family history diabetes [AOR = 0.38 (95%CI: 0.17–0.82), p = 0.014].

**Conclusion:**

More than half of the studied population had inadequate knowledge of diabetes complications. Female gender, rural dwellers, and low education level were factors positively associated with inadequate knowledge of diabetes complications. A multisectoral approach is needed, where the government of Ghana together with other sectors of the economy such as the health, education and local government sectors work collaboratively in the development of locally tailored diabetes education programmes to promote healthy self-care behaviours relevant for the prevention of diabetes and its complications.

## Introduction

Diabetes mellitus (DM) is a complex disease that affects many organ systems, leading to concerns about deteriorating population health status and ever-increasing healthcare expenditure [1, 2]. It is recognised as one of the leading causes of death and disability worldwide [3]. In 2016, DM was the seventh leading cause of mortality globally with a projected 1.6 million deaths directly caused by diabetes [4]. As of 2019, 463 million people had diabetes worldwide. This figure is estimated to increase to 700.2 million by 2045 [5]. Around 80% of the global surge is expected to happen in Africa [6]. Diabetes prevalence studies in Ghana have recorded a marked increase. The earliest study in the 1960s reported 0.2% prevalence in a population of Ghanaian men in Ho [7]. The crude prevalence of diabetes among the general population was 6.3% in the late 1990s in Accra and the age-adjusted prevalence and impaired glucose tolerance were 6.1% and 10.7% respectively [8]. Type 2 diabetes mellitus (T2DM) accounts for 90% of all cases [5].

Uncontrolled diabetes can lead to life-threatening complications including both micro and macrovascular complications such as cardiovascular diseases, retinopathy, end-stage renal disease, and neuropathy [9]. The prevalence of diabetes and its complications is rising exponentially in China [10], Ireland [11] and northern Africa [12]. In Ghana, the prevalence of hypertention, retinopathy and neuropathy were 96.2%, 58.6%, and 60.5% respectively [13]. Diabetes-related health outcomes, treatment choices, care needs, and associated expenditures are exacerbated by the presence of these complications existing in addition to T2DM [14]. Policymakers, healthcare professionals, and persons with diabetes are increasingly focused on improving diabetes management, including achieving glycaemic control, lipid and blood levels, and blood pressure targets, which have been shown to decrease some diabetes complications [2]. Although many of these efforts have focused on improving diabetes management performance of healthcare providers [2], diabetes-related knowledge of patients is also critically important to achieve diabetes care targets and minimise complications.

Adequate knowledge is a major component of diabetes management. Increasing patients’ knowledge regarding DM and its complications has significant benefits concerning adherence to treatment and reducing complications [15, 16]. Several studies have been done on knowledge and management of diabetes across the globe [17–22]. Although some studies have been conducted on diabetes-related knowledge in Ghana, a few have focused on assessing knowledge on complications in particular [23, 24]. A recent study conducted among persons living with T2DM at a district hospital in Ghana reported low level of knowledge on diabetes complications where only 13.1% had adequate knowledge [20]. Despite these studies, most Ghanaians are still less knowledgeable about DM and its associated complications [25].

People with inadequate knowledge and low health literacy on diabetes complications will likely have suboptimal glycaemic control and increased risk of getting complications as they often have challenges understanding and following medical instructions [26]. Knowledge of diabetes complications is relevant for early recognition of the warning signs and symptoms necessary for the planning and implementation of appropriate preventive interventions to avert or delay complications [27]. There is limited data on knowledge of chronic complications of DM within the Ghanaian context [20]. Such data is important to policymakers and healthcare professionals for the development and implementation of appropriate clinical and public health strategies aimed at controlling and preventing the consequences of the disease. Therefore, this study aimed to evaluate the knowledge of chronic complications of diabetes among persons living with type 2 diabetic mellitus in northern Ghana.

## Methodology

### Study design

A descriptive cross-sectional design was used to determine the knowledge of diabetes complications among persons living with T2DM in northern Ghana.

### Study setting

This study was conducted at three government hospitals namely; Tamale Teaching Hospital, Tamale West, and Central Hospitals in the northern region of Ghana. The Tamale teaching hospital serves as a referral center for all the five regions of the north and some parts of the Brono and Ahafo regions. The Tamale west, and central hospitals are secondary level facilities and serve as referral centers for some districts in the region. These hospitals are located in the Tamale metropolis, the administrative capital. Healthcare in the metropolis is provided by public/private hospitals, health centers, clinics and traditional medicine clinics/traditional healers. The metropolis is predominantly urban, however, some residents in adjoining rural communities seek medical services in Tamale. Available health facilities in rural communities include clinics, health centers, and community health planning and services (CHPS) providing primary health services [28]. Due to financial challenges, and lack of equitable health services, most rural residents including non-affluent urban dwellers often resort to traditional healers or herbalist for their healthcare needs including treatment for diabetes [29].

### Study population

The study population comprised all patients with T2DM registered with the outpatient diabetes clinics at the three health facilities. The population of patients with T2DM in these hospitals was estimated at 1800 for three months from clinical data. Patients who sought routine medical services at the outpatient endocrinology clinics of the selected hospitals were identified during the study period. Participants were included in the study if they had; a diagnosis of T2DM for at least one year, were aged 18 years and older, and attended the clinics for at least one year. Patients with Type 1 DM and patients who had mental instability were excluded.

### Sampling and sample size

The consecutive sampling technique was employed to recruit participants who reported to the endocrinology clinics for routine medical services. The sample frame for the number of persons with T2DM who visit the clinics of the selected hospitals was estimated at 1800 for three months. The sample size for the study was calculated using the formula for sample size determination by Yamane [30].

$$n=\frac{N}{1+Ne^{2}}$$

Where ***n*** is required sample size.

***N*** is the total population size which is 1800.

***e*** is acceptable sampling error (0.05) at 95% Confidence Interval

By substitution:

$$n=\frac{1800}{1+1800({0.05)}^{2}}\;n=327$$

Hence, the sample size for the study = **327** participants. A 5% non response rate was calculated, increasing the sample size to 344.

### Instrument

A structured instrument was used to obtain information from all the study participants. The questionnaire was developed from the review of related litereature in the subject area [20, 31, 32]. It was divided into two sections. The first section involved questions that elicited information on socio-demographic variables of participants including; age, gender, educational level, occupation, duration of DM, type of treatment, residence, duration and regularity of clinic visit, presence of complication and family history of diabetes. Information on complications was obtained by self-report and subsequent review of participants medical records for confirmatory diagnosis. The second section included questions that assessed participants knowledge on complications of diabetes and the kind of complications they knew. Each question had one correct answer from three answer choices. The answer choices for each question were “Yes”, “No” and “Don’t Know”. The “Don’t know” option was included to minimise guesswork. The participants’ responses were analysed as either correct or incorrect. One point was awarded for each right response, and zero for each incorrect or “Don’t know response”. The mean knowledge score was calculated and knowledge was categorised as either inadequate or adequate. Participants who had a score of (<Mean– 1 SD) were coded as having inadequate knowledge and scores that corresponded to (>Mean + 1 SD) were considered as adequate knowledge [33].

### Reliability and validity

The questionnaire was pre-tested among 25 patients with T2DM to ascertain its content and clarity. Reliability coefficients ranging from 0.00 to 1.00, with higher coefficients indicating higher levels of reliability was used to determine the validity and the reliability of the questionnaire. The reliability coefficients for all the questions were 0.901.

### Data collection

The data collection team comprised of three nurses who were trained as research assistants and supervised by the principal investigator. Participants were approached during the endocrinology clinic days of various hospitals to collect data. The objectives and procedures of the study were explained to participants both in English and in the local dialect. Written informed consent was obtained from each participant. The questionnaires were self-administered to participants who could read and write in English. Participants who could not read nor write in English were assisted by the research assistants in terms of translating the questions into their respective native languages. The research assistants received training on how to translate the questions to ensure uniformity of interviews. The data collection spanned from September to November 2018.

### Data analysis

Out of the 344 participants invited, 14 declined participation and 10 participants’ medical records were not available at the time of data collection. Therefore, a total of 320 patients participated in the study, resulting in a 93.0% response rate. The data entry and analysis were performed using IBM statistical package for social science (SPSS) version 23. Descriptive statistics such as frequencies and percentages were used. Bivariate logistic regression was used to model the factors associated with participants knowledge on diabetes complications. Sex, age, education, monthly income, residence, duration of diabetes, type of treatment, family history of diabetes, duration of clinic visit, smoking status, were variables statistically significant in the bivariate logistic regression model and were considered for the multivariable logistic model to account for confounding effect. Both the bivariate and multivariate logistic regression model were used to test the association between categorical variables to predict factors associated with knowledge of diabetes complications. A *P*-value of less than 0.05 was considered statistically significant.

### Ethical consideration

Ethical approval was obtained from the ethics review committee of the Kwame Nkrumah University of Science and Technology (CHRPE/AP/576/18). Permission was sought from the management of the various hospitals. Confidentiality and anonymity of participants were ensured as no personal identifiers were used in the study. Participation was voluntary and they were free to participate or not and if they declined, it would not affect their regular care.

## Results

### Socio-demographic characteristics of respondents

As indicated in Table 1, the mean (SD) age of participants was 57.5 (11.67) years. The majority of participants, 68.4% were women, 60.9% had no formal education and 43.4% were self-employed. More than three-fourth of the participants (79.7%) had family support. About two-thirds (70.3%) earned an income of less than GHS 500 each month. Nearly 74% lived in an urban setting and 37.8% had diabetes for a period of 1 to 3 years. The majority of participants (74.4%) were under oral hypoglycaemic treatment. A significant proportion of participants (40%) had medically confirmed diabetes complication. More than half (55.6%) of participants visited the clinic every 2 months and 43.8% visited the clinic monthly.

**Table 1:** Socio-demographic characteristics.

Variable		Frequency (N = 320)	Percent (%)
**Gender**	Male	101	31.6
	Female	219	68.4
**Mean age ± SD**	57.52 **±** 11.67		
**Age**	< 50 years	78	24.4
	50–59 years	90	28.1
	60–69 years	93	29.1
	70+ years	59	18.4
**Educational level**	Tertiary	49	15.3
	Senior high school	31	9.7
	Junior high school	22	6.9
	Primary school	23	7.2
	No formal education	195	60.9
**Occupation**	Private Sector employment	25	7.8
	Public Sector employment	55	17.2
	Self-employed	139	43.4
	No employment	101	31.6
**Marital status**	Single	16	5.0
	Married	216	67.5
	Divorced	29	9.1
	Widowed	59	18.4
**Family Support**	Yes	255	79.7
	No	65	20.3
**Religion**	Christian	83	25.9
	Muslim	237	74.1
**Monthly income**	<GHS 500	225	70.3
	GHS 500–1000	64	20.0
	GHS 1000–2000	19	5.9
	GHS 2000–3000	10	3.1
	>GHS 3000	2	0.6
**Residence**	Urban	236	73.7
	Rural	84	26.3
**Duration of diabetes**	1–3 years	121	37.8
	4–6 years	95	29.7
	7–9 years	44	13.7
	> = 10 years	60	18.8
**Type of treatment**	Oral hypoglycaemic	238	74.4
	Insulin	33	10.3
	Both oral and insulin	49	15.3
**Medically confirmed diabetic complications**	Yes	128	40.0
	No	185	57.8
	Not sure	7	2.2
**Family history diabetes**	Yes	128	40.0
	No	126	39.4
	Don’t know	66	20.6
**Smoking status**	Yes	15	4.7
	No	305	95.3
**Duration of diabetes clinic visit**	1–2 years	104	32.5
	3–4 years	69	21.6
	5–6 years	60	18.7
	>6 years	87	27.2
**Regularity of clinic visit**	Monthly	140	43.8
	Every 2 months	178	55.6
	Every 3 months	2	0.6

### Knowledge of participants on complications of diabetes mellitus

The overall level of knowledge revealed that less than half, 45.9% of participants had adequate knowledge of diabetes complications, and the remaining 54.1% had inadequate knowledge.

As indicated in Table 2, the knowledge of diabetes complications revealed that only 57.8% of participants knew that diabetes could cause damage to the kidney. Most of them, 78.8% knew that one could develop neuropathy as a result of diabetes. Less than half, 45.0% and 49.4% had knowledge that diabetes could cause retinopathy (blurred vision) and hypertension respectively. Around 58.8% and 74.4% of the participants knew that heart diseases and diabetic foot ulcers are complications of diabetes. Only 26.3% of the participants were aware of hypo-sexual dysfunction as a complication of diabetes.

**Table 2:** Knowledge of complications of diabetes mellitus.

Knowledge of diabetes complications		Frequency (N = 320)	Percent (%)
**Kidney diseases**	Yes	185	57.8
****	No	50	15.6
	Don't know	85	26.6
**Neuropathy**	Yes	252	78.8
	No	33	10.3
	Don't know	35	10.9
**Retinopathy(blurred vision)**	Yes	144	45.0
	No	148	46.2
	Don't know	28	8.8
**Heart diseases**	Yes	188	58.8
	No	57	17.8
	Don't know	75	23.4
**Diabetic foot ulcers**	Yes	238	74.4
	No	35	10.9
	Don't know	47	14.7
**Hypo-sexual dysfunction**	Yes	84	26.3
	No	136	42.5
	Don't know	100	31.2
**Hypertension**	Yes	158	49.4
	No	54	16.9
	Don't know	108	33.7

### Factors influencing knowledge of complications of diabetes

In Table 3, the multivariable logistic regression revealed that women were 71% less likely to have adequate knowledge of diabetes complication compared with men [AOR = 0.29 (95%CI: 0.14–0.56), p<0.001]. Participants who were aged between 60 and 69 years were 55% less likely to have adequate knowledge compared to those who were less than 50 years [AOR = 0.45 (95%CI:0.20–0.99), p = 0.049]. Participants with primary education and those without formal education were 87% and 84% less likely to have adequate knowledge of diabetes complications compared with their counterparts who had tertiary education [AOR = 0.13 (95%CI: 0.03–0.51), p = 0.004]; [AOR = 0.16 (95%CI: 0.05–0.50), p = 0.002] respectively. Moreover, participants who lived in rural settings were 50% less likely to have adequate knowledge of diabetes complications compared to those who lived in urban areas [AOR = 0.50 (95%CI: 0.27–0.95), p = 0.033]. Similarly, participants who were unaware of their family history of diabetes were 62% less likely to have adequate knowledge of diabetes complications compared to those with a family history of diabetes [AOR = 0.38 (95%CI: 0.17–0.82), p = 0.014]. Moreover, participants who attended the diabetes clinic for more than 6 years were 14 times more likely to have adequate knowledge of diabetes complications [AOR = 14.43 (95%CI: 3.26–60.26), p<0.001].

**Table 3:** Factors influencing knowledge of complications of diabetes.

Variables		IK n = 173	AK n = 147	COR [95% CI]	p-value	AOR [95% CI]	p-value
**Gender**							
	Male	36 (20.8)	65 (44.2)	Reference		Reference	
	Female	137 (79.2)	82 (55.8)	0.33 [0.20–0.54]	**<0.001**	0.29 [0.14–0.56]	**<0.001**
**Age group**							
	< 50 years	37 (21.4)	41 (27.9)	Reference		Reference	
	50–59 years	42 (24.3)	48 (32.6)	1.03 [0.56–1.89]	0.921	1.04 [0.48–2.23]	0.926
	60–69 years	59 (34.1)	34 (23.1)	0.52 [0.28–0.96]	**0.037**	0.45 [0.20–0.99]	**0.049**
	70+ years	35 (20.2)	24 (16.3)	0.62 [0.31–1.23]	0.169	0.71 [0.30–1.67]	0.429
**Education**							
	Tertiary education	9 (5.2)	40 (27.2)	Reference		Reference	
	Senior high school	12 (6.9)	19 (12.9)	0.36 [0.13–0.99]	**0.048**	0.38 [0.11–1.37]	0.139
	Junior high school	8 (4.6)	14 (9.5)	0.39 [0.13–1.21]	0.106	0.26 [0.06–1. 13]	0.073
	Primary school	15 (8.7)	8 (5.4)	0.12 [0.04–0.37]	**<0.001**	0.13 [0.03–0.51]	**0.004**
	No formal education	129 (74.6)	66 (44.9)	0.12 [0.05–0.25]	**<0.001**	0.16 [0.05–0.50]	**0.002**
**Occupation**							
	Private Sector employment	8 (4.6)	17 (11.6)	Reference		Reference	
	Public Sector employment	20 (11.6)	35 (23.8)	0.82 [0.30–2.25]	0.705	1.10 [0.30–4.04]	0.891
	Self-employed	85 (49.1)	54 (36.7)	0.30 [0.12–0.74]	**0.009**	0.95 [0.26–3.38]	0.932
	No employment	60 (34.7)	41 (27.9)	0.32 [0.13–0.81]	**0.017**	0.96 [0.26–3.50]	0.945
**Marital status**							
	Single	11 (6.4)	5 (3.4)	Reference			
	Married	113 (65.3)	103 (70.1)	2.00 [0.67–5.97]	0.211		
	Divorced	15 (8.7)	14 (9.5)	2.05 [0.57–7.41]	0.272		
	Widowed	34 (19.7)	25 (17.0)	1.62 [0.50–5.25]	0.423		
**Family Support**							
	Yes	133 (76.9)	122 (83.0)	Reference			
	No	40 (23.1)	25 (17.0)	0.68 [0.39–1.19]	0.177		
**Religion**							
	Christian	45 (26.0)	38 (25.8)	Reference			
	Muslim	128 (74.0)	109 (74.2)	1.01 [0.61–1.66]	0.974		
**Monthly income**							
	<GHS 500	134 (77.5)	91 (61.9)	Reference		Reference	
	GHS 500–1000	31 (17.9)	33 (22.4)	1.57 [0.90–2.74]	0.114	0.68 [0.30–1.52]	0.351
	GHS 1000–2000	5 (2.9)	14 (9.5)	4.12 [1.43–11.84]	**0.009**	1.04 [0.26–4.10]	0.959
	GHS 2000–3000	3 (1.7)	7 (4.8)	3.43 [0.86–13.64]	**0.079**	0.47 [0.08–2.85]	0.409
**Residence**							
	Urban	116 (67.1)	120 (81.6)	Reference		Reference	
	Rural	57 (32.9)	27 (18.4)	0.46 [0.27–0.77]	**0.003**	0.50 [0.27–0.95]	**0.033**
**Duration of diabetes**							
	1–3 years	75 (43.4)	46 (31.3)	Reference		Reference	
	4–6 years	53 (30.6)	42 (28.6)	1.29 [0.75–2.23]	0.358	0.73 [0.29–1.88]	0.519
	7–9 years	21 (12.1)	23 (15.6)	1.79 [0.89–3.58]	0.103	0.23 [0.05–1.03]	0.055
	> = 10 years	24 (13.9)	36 (24.5)	2.45 [1.30–4.61]	**0.006**	0.25 [0.06–1.09]	0.065
**Type of treatment**							
	Oral hypoglycaemic	138 (79.8)	100 (68.0)	Reference		Reference	
	Insulin	16 (9.2)	17 (11.6)	1.47 [0.71–3.01]	0.304	0.99 [0.40–2.44]	0.987
	Both oral and insulin	19 (11.0)	30 (20.4)	2.18 [1.16–4.09]	**0.015**	1.16 [0.51–2.60]	0.726
**Confirmed diabetic complications**							
	Yes	75 (43.3)	53 (36.0)	Reference			
	No	93 (53.8)	92 (62.6)	1.40 [0.88–2.20]	0.147		
	Not sure	5 (2.9)	2 (1.4)	0.57 [0.11–3.02]	0.506		
**Family history of diabetes**							
	Yes	60 (34.7)	68 (46.3)	Reference		Reference	
	No	70 (40.4)	56 (38.1)	0.70 [0.43–1.16]	0.167	0.71 [0.38–1.35]	0.303
	Don’t know	43 (24.9)	23 (15.6)	0.47 [0.25–0.87]	**0.017**	0.38 [0.17–0.82]	**0.014**
**Smoking/Alcohol status**							
	Yes	13 (7.5)	2 (1.4)	Reference		Reference	
	No	160 (92.5)	145 (98.6)	5.89 [1.31–26.55]	**0.021**	6.23 [0.99–39.10]	0.051
**Duration of clinic visit**							
	1–2 years	71 (41.0)	33 (22.4)	Reference		Reference	
	3–4 years	43 (24.9)	26 (17.7)	1.30 [0.69–2.46]	0.419	1.38 [0.57–3.33]	0.476
	5–6 years	29 (16.8)	31 (21.1)	2.30 [1.20–4.42]	**0.012**	2.17 [0.74–6.36]	0.156
	>6 years	30 (17.3)	57 (38.8)	4.09 [2.23–7.48]	**<0.001**	14.43 [3.26–60.26]	**<0.001**
**Regularity clinic visit**							
	Monthly	80 (46.2)	60 (40.8)	Reference			
	Every 2 months	92 (53.2)	86 (58.5)	1.25 [0.80–1.94]	0.333		
	Every 3 months	1 (0.6)	1 (0.7)	1.33 [0.08–21.75]	0.840		

IK: Inadequate Knowledge, AK: Adequate Knowledge

## Discussion

This study aimed to assess the knowledge of complications of diabetes among persons living with T2DM in northern Ghana. Adequate knowledge of DM and its complications is necessary for diabetes self-management. It is a prerequisite for reduction of unhealthy behaviours and subsequent prevention and/or reduction of the development of complications associated with the disease. In this study, it was observed that a substantial proportion of the study participants (40%) reported having one or more chronic complications of DM. This implies that, these patients probably coud not control their diabetes leading to complications. Healthcare providers need to take pragmatic plans and shift priorities to focus on patients with complication(s) for the prevention of disability and death. This finding also brings to the fore the need for the establishment of national diabetes programmes to provide prevention and control strategies, considering the huge economic burden associated with treating DM and its complications [34].

Generally, the majority of participants (54.1%) in this study had inadequate knowledge of diabetes complications. This may be explained by the low educational and socioeconomic status of the current study population. It is worth noting that almost two-thirds of the participants had no formal education. This is a matter of concern, as there are no available culturally and linguistically appropriate diabetes education resources in the Ghanaian context [35]. Consequently, these patients may not be able to communicate effectively with health professionals due to low literacy. This could pose a challenge to effective counseling on diabetes and its associated complications. The present study finding is consistent with previous studies conducted in Ghana [20], Ethiopia [36], and India [37], whereby most participants had inadequate knowledge of diabetes complications. Conflicting findings were reported in Nigeria where 90.5% of T2DM patients had adequate knowledge of diabetes complications [27]. The variation could be due to the difference in socioeconomic and cultural characteristics which have been established to have an influence on patients knowledge [36].

Concerning participants knowledge on specific complications, the most common complications known by participants were; neuropathy followed by diabetic foot ulcer, heart diseases, kidney disease, hypertension, retinopathy and hypo-sexual dysfunction. The disparity on knowledge of different complications could imply that participants might have experienced them. A previous study done in Ghana by Obirikorang et al. [20] found out that, participants knew diabetic foot (51.5%), hypertension (35.4%), neuropathy (29.2%), hypoactive sexual arousal (25.4%), arousal disorder (21.5%), retinopathy (17.7%), heart disease (9.2%), and nephropathy (5.4%) as the most common DM complications. Another study by Konduru, Ranjan, Karthik and Shaik [38] revealed that eye complications (69%) was commonly known by diabetic patients followed by cardiac complications (51%), and central nervous system complications (28%). The current study also differ from a study conducted in Malaysia where the majority of participants (61.25%) mentioned that diabetic foot ulcer is the most common complications of diabetes. However, heart disease (27.75%), kidney disease (38.25%), eye disease (32.50%), and stroke (20%) were also reported by the participants as the common complications of diabetes [39]. This disparity between our study and prior studies may be attributed to the variation in the diabetes education provided to participants. Moreover, variations in culture, race, and ethnicity among the populations may influence the pattern of knowledge on diabetes complications [36].

Another interesting finding of this study is the positive association between educational status and the level of participants’ knowledge of diabetes complications. Participants with tertiary education had significantly higher knowledge than their counterparts with low level of education. This is in line with findings reported in a study done among T2DM patients visiting the diabetes clinic at Sampa Government Hospital in Ghana where there was a relationship between the level of education and the degree of knowledge on diabetic complications [20]. This is not surprising as participants who completed tertiary education may have attended workshops, conferences, seminars and health talks on health-related issues. Moreover, they may browse the internet for more information on DM to enhance their knowledge [36].

It is worth noting that, there was a positive association between gender and knowledge of diabetes complications in this study. Women were less likely to have adequate knowledge compared with men. Similar findings were observed in previous studies conducted in Ethiopia [36], Pakistan [40], India [37] and Ghana [20] where men were found to have more knowledge on diabetes complications than women. This finding could be attributed to cultural influence which allows males to spend most of their time outside the home, attend different meetings and conferences which might have provided them with the opportunity to obtain more information on diabetes than their female counterparts who are always at home [36]. We recommend the implementation of targeted educational programmes to enhance the knowledge of women.

A previous study conducted among patients with T2DM in Pakistan indicated that urban settlers were more knowledgeable on diabetes complications than their colleagues residing in the rural areas [41]. This finding is congruent with the present study finding where urban dwellers compared with rural settlers had adequate knowledge of diabetes complications. A similar finding was observed by Hoque, Islam, Khan, Aziz, and Ahasan [31]. Participants from rural areas were probably less educated or may have had less regular diabetes clinic visits due to financial challenges, as there is lack of equitable access to facilities particularly in rural communities in Ghana. Efforts at promoting education and health literacy on diabetes and its associated complications among rural inhabitants need to be intensified by government and healthcare providers. Community-wide education programmes is recommended to raise awareness of modifiable risk factors of diabetes and its complications which will have benefits beyond diabetes [5].

### Limitations of the study

The sample population might not reflect the overall T2DM patients in Ghana. The patients who seek care in traditional medicine clinics or private hospitals or those who did not seek care regularly in these hospitals might not be included in this study. Thus, the findings cannot be generalized. The cross-sectional design does not establish causality though it demonstrates association between variables. Notwithstanding, the current study provides vital information on the level of knowedge of long-term complications among participants that can form the basis for patient education.

## Conclusion

More than half of the studied population had inadequate knowledge of diabetes complications. The most common complications of DM known by participants were; neuropathy followed by diabetic foot ulcers, heart diseases, kidney disease, hypertension, retinopathy (blurred vision), and hypo-sexual dysfunction. Female gender, rural dwellers, and low educational level were the factors positively associated with inadequate knowledge of diabetes complications. A multisectoral approach is needed, where the government of Ghana together with other sectors such as the health, education and local government sectors work collaboratively in the development of locally tailored diabetes education programmes to promote healthy self-care behaviours relevant for the prevention of diabetes and its complications. Healthcare providers need to intensify education on diabetes, treatment, and complications utilising linguistically and culturally appropriate educational resources to enhance patients' knowledge.

## Supporting information

S1 Dataset (XLSX)

## Abbreviations

DM: Diabetes mellitus
T2DM: Type 2 diabetes mellitus
BMI: Body mass index
WC: Waist circumference
WHO: World health organization
SD: Standard deviation
SHS: Senior high school
JHS: Junior high school
CHPS: Community health planning and services
